# Effect of *Lactobacillus reuteri* Postbiotics on Growth Performance and Intestinal Health of *Escherichia coli*-Infected Broilers

**DOI:** 10.3390/ani16010082

**Published:** 2025-12-27

**Authors:** Changwu Li, Jiarou Fan, Yafei Zhang, Yu Zhang, Jiakun Yan, Peng Li, Shuangshuang Guo, Binying Ding

**Affiliations:** 1 Hubei Key Laboratory of Animal Nutrition and Feed Science, Wuhan Polytechnic University, Wuhan 430023, China; lichangwu105@163.com (C.L.); fan1jiarou@163.com (J.F.); 13598432475@163.com (Y.Z.); 13781843612@163.com (Y.Z.); yanjk16@whpu.edu.cn (J.Y.); dbying7471@126.com (B.D.); 2 Engineering Research Center of Feed Protein Resources of Agricultural By-Products in Ministry of Education, Wuhan Polytechnic University, Wuhan 430023, China

**Keywords:** *Lactobacillus reuteri*, postbiotics, *Escherichia coli*, intestinal health, broiler chicken

## Abstract

Avian pathogenic *Escherichia coli* (*E. coli*) impairs poultry production and causes substantial economic losses. This study investigated the effects of *Lactobacillus reuteri* postbiotics (LR) on growth performance and intestinal health of broiler chickens challenged with *E. coli*. A total of 180 one-day-old Arbor Acres+ broilers were allocated into three groups (six replicates per group and 10 chicks each replicate): CTR, control group; *E. coli*-infected group, orally challenged with a mixture of *E. coli* O_1_, O_2_, and O_78_ at a dose of 10^9^ CFU/mL; LR *+ E. coli*-infected group, challenged with *E. coli* and fed a basal diet supplemented with 100 mg/kg LR. The results showed that dietary LR significantly improved the average daily gain (ADG) in the LR + *E. coli* group compared to the *E. coli*-infected group from days 1 to 18 (*p* < 0.05). However, no significant differences in average daily feed intake (ADFI) or feed conversion ratio (FCR) were observed among the CTR, *E. coli*, and LR + *E. coli* groups. Infection with *E. coli* led to lower total antioxidant capacity in jejunum and activity of total superoxide dismutase in ileum. Moreover, dietary LR significantly alleviated the down-regulation of *Mucin2* and *Aquaporin-3* gene expression in jejunum and ileum caused by *E. coli* infection and up-regulated the gene expression of *Claudin-1* and *Zonula occludens* 1 in the ileum. In addition, dietary LR treatment led to the up-regulation of *interleukin-10* mRNA transcripts in the jejunum. Further analysis demonstrated that dietary supplementation with LR reshaped the ileal flora of birds challenged with *E. coli* via elevating the relative abundance of *Romboutsia* and *Bacteroidota*, while reducing the abundance of *Candidatus_Arthromitus* and *Escherichia-Shigella.* In conclusion, dietary LR supplementation improved the expression of intestinal barrier and anti-inflammatory genes and reshaped the intestinal flora in *E. coli*-infected broilers.

## Simple Summary

*Lactobacilli*, among the most common probiotics in the broiler intestinal tract, play a key role in maintaining intestinal homeostasis and improving growth performance. In this study, we observed that dietary *Lactobacillus reuteri* postbiotics ameliorated *E. coli*-induced mild jejunal barrier damage by up-regulating tight junction proteins and anti-inflammatory cytokines. Dietary addition of *Lactobacillus reuteri* postbiotics increased the relative abundance of *Romboutsia* and decreased the relative abundance of *Escherichia-Shigella* in the ileum. *Lactobacillus* postbiotics showed potential benefits to the intestinal health of *E. coli*-infected broilers.

## 1. Introduction

*Escherichia coli* is a common pathogen that causes significant losses for the global poultry industry [1]. The *E. coli* usually appears in dirty waterlines, dead birds, contaminated feed, and infected chicks (breeder and offspring). The vertical and horizontal transmission of *E. coli* causes several diseases, low growth performance, antimicrobial resistance, food safety, and the emergence of antibiotic-resistant bacteria [2]. At an early age, avian pathogenic *E. coli* challenge can cause losses in body weight gain, feed intake, and feed efficiency, along with liver damage by increasing the mRNA level of inflammatory genes and decreasing antioxidant capacity [3]. The *E. coli* stimulates production of several proinflammatory cytokines such as interleukin-1beta (IL-1β), interferon-gamma (IFN-γ), and tumor necrosis factor-alpha (TNF-α) [4]. Moreover, oxidative damage, intestinal permeability, and gut microbiota are susceptible under *E. coli* infection [5]. Thus, preventing and controlling the immune and oxidative stress induced by *E. coli* has become an urgent issue.

Various antibiotics are used to prevent and control *E. coli* infection [6], which can lead to public health concerns and antimicrobial resistance [7]. To avoid these risks, numerous alternatives such as probiotics, prebiotics, enzymes, short-chain fatty acids, antimicrobial peptides, essential oils, and organic acids have been used for improving animal health [8]. In the poultry industry, probiotics (or direct-fed microbials) and prebiotics have been used to improve the growth performance and intestinal microbiota in broilers [9]. While probiotics are beneficial for animal health, their efficacy can be inconsistent, particularly over prolonged periods. Postbiotics are the preparations of inanimate microorganisms and/or their fermentation components [10]. Postbiotics obtained from non-viable microorganisms are less likely to cause bacteremia or fungemia than probiotics [11]. Additionally, postbiotics from *Lacticaseibacillus rhamnosus*, *Limosilactobacillus reuteri*, and *Bifidobacterium animalis* subsp. *Lactis Bb12* can protect macrophages against crystalline silica-induced cytotoxicity and suppress IL-1β activation [12]. Yeast postbiotics can alleviate diarrhea and improve gut health performance in weaned piglets [13]. However, inconsistent results of dietary supplementation with postbiotics on gut mucosal barrier, morphology, microbiota, and subsequent growth performance were demonstrated [14,15]. Recent studies showed that postbiotics exert beneficial effects on improving poultry health under obvious pathogen challenge [14,16].

*Lactobacilli*, the most common beneficial bacteria in broiler intestine, play a key role in maintaining intestinal homeostasis, improving growth performance, and immunity. *Lactobacilli* supplementation can re-establish proper microbial balance by producing lactate, propionate, and butyrate in the cecum of chickens [17]. Postbiotics derived from *Bifidobacterium lactis* and *Lactobacillus reuteri* can effectively alleviate liver injury and colitis susceptibility by modulating enterohepatic circulation of bile acids and gut microbial composition [18]. However, there is a lack of direct evidence regarding the effects of *Lactobacillus reuteri* postbiotics (LR) on young broilers under *E. coli* infection.

The present study aimed to examine the impact of postbiotics from *Lactobacillus reuteri* and its metabolites on intestinal health of chicks infected with *E. coli*. The effects of LR were investigated through in vivo assessments, including growth performance, plasma biochemical indices, intestinal barrier function, antioxidant function, inflammatory response, and ileal microbiota of broiler chickens.

## 2. Materials and Methods

### 2.1. Experimental Animals, Diets, and Design

All animal procedures used in this study were approved by the Institutional Animal Care and Use Committee of Wuhan Polytechnic University (Number: WPU20230711). A total of 180 one-day-old Arbor Acres+ broilers were allocated into three groups (6 replicates per group and 10 chicks each replicate): CTR, control group; *E. coli*-treated, co-infected with *E. coli* O_1_, O_2_, and O_78_ at a dose of 10^9^ CFU/mL; LR + *E. coli*-treated, infected with *E. coli* and supplemented with 100 mg/kg LR. The experiment lasted for 28 days, and LR was added throughout the trial. Birds in the control (CTR) and *E. coli*-challenged groups were fed the basal diet, while those in the LR + *E. coli* group received the basal diet supplemented with 100 mg/kg LR. The LR was provided by Hubei Blue Valley Microbial Technology Co., Ltd. (Yichang, Hubei, China). The LR contained the following components: bacterial protein ≥ 27%, *L. reuteri* ≥ 10^10^ CFU/g, bacteriocin ≥ 224 mg/g, and mussel mucin ≥ 5 mg/g. The maize–soybean meal basal diet used in the experiment was formulated according to the NY/T33-2004 (feeding standard of China) recommendations for broilers. The dietary formula and nutritional levels are shown in Table 1. All broilers were raised in wire cages, with free access to water and feed. Birds were kept in a controlled room temperature at 35 °C in the first week, and then, the temperature decreased by 2–3 °C per week until maintained at 22 °C. A 24 h light regime was performed throughout the trial.

### 2.2. Establishment of Escherichia coli Model

The microbiota of broilers achieved stability at 21 days of age [19]. To induce mild intestinal injury, from days 12 to 18, broilers in the *E. coli* and LR + *E. coli*-treated groups were continuously orally injected with 2 mL of a mixture of *E. coli* O_1_, O_2_, and O_78_ at a concentration of 1.0 × 10^9^ CFU/mL. The *E. coli* O_1_, O_2_, and O_78_ was cultured individually until the stationary phase, with optical density at 2.30–2.50. The viable bacterial number was confirmed by plate count. The mixture of *E. coli* consisted of *E. coli* O_1_, O_2_, and O_78_ with a ratio of 3:3:4. Birds in the CTR group were given the same volume of saline. The *E. coli* O_1_, O_2_, and O_78_ were kindly provided by the State Key Laboratory of Animal Nutrition and Feeding, China Agricultural University.

### 2.3. Sample Collection

On day 19, two birds per replicate were randomly selected after feed deprivation and weighed, and blood samples were collected from the wing vein. The blood samples were allowed to clot at room temperature for 2 h. Serum was separated by centrifugation at 3000 g for 15 min at 4 °C, then stored at −80 °C for further analysis. Subsequently, birds were euthanized by cervical dislocation and slaughtered. Samples from the middle sections (approximately 1 cm) of the jejunum and ileum were fixed in 4% paraformaldehyde and embedded in paraffin. Another approximately 5 cm of the jejunum and ileum were carefully collected and washed with PBS. Digesta from the ileum was collected and stored at −80 °C for microbial composition analysis.

### 2.4. Measurements of Growth Performance

On days 19 and 28, the birds were feed-deprived for 8 h, and then the feed intake and body weight (BW) of the birds in each replicate were measured. The average daily feed intake (ADFI), average daily gain (ADG), feed conversion ratios (FCRs, feed intake/BW gain), and mortality of the birds were calculated for days 1–18, 19–28, and 1–28, respectively.

### 2.5. Serum Diamine Oxidase Analysis

Diamine oxidase (DAO) in serum was determined using commercially available assay kits (A088, A044; Nanjing Jiancheng Bioengineering Institute, Nanjing, Jiangsu, China) according to the manufacturer’s instructions.

### 2.6. Intestinal Morphology

Jejunal and ileal tissues were sectioned (5 μm thickness), stained with hematoxylin and eosin (HE), and determined using the Olympus BX-41TF microscope with image analysis software (Olympus Corporation, Tokyo, Japan). Villus height (VH) and crypt depth (CD) were blindly measured. Ten villi from each section were randomly selected for measurements, and VH to CD ratio (VH/CD) was then calculated.

### 2.7. Intestinal Histologic Scoring

The intestinal sections with HE staining were used for histologic scoring. The scoring system from 0 to 4 was described by Dameanti et al. (2023) [20]. Score 0, no change/normal; score 1, minimal damage (edema and shortening of villi); score 2, minor damage (villi are lightly torn/dulled, and goblet cells proliferate); score 3, moderate damage (infiltration of inflammatory cells); score 4, severe damage (necrosis).

### 2.8. Measurements of Intestinal Antioxidant Status

Approximately 1 g of jejunal and ileal tissues were homogenized in 9 mL of ice-cold saline and then centrifuged at 3500 *g* for 15 min at 4 °C. Supernatants were collected for measurements of antioxidant status. The levels of total antioxidant capacity (T-AOC), total superoxide dismutase (T-SOD), and malondiadehyde (MDA) were determined using commercial kits (Nanjing Jiancheng Bioengineering Institute, Nanjing, China).

### 2.9. Expression of Intestinal Barrier and Immunity-Related Genes

Total RNA from the jejunum and ileum was extracted using the TRIzol^TM^ Reagent kit (Invitrogen, Carlsbad, CA, USA) according to the manufacturer’s instructions. The concentration of RNA and its optical density 260/280 value were quantified using a NanoDrop^®^ ND-2000 UV-VIS spectrophotometer (Thermo Scientific, Wilmington, DE, USA). The cDNA was obtained from 1 µg RNA using a PrimeScript^®^ RT reagent Kit with gDNA Eraser (Takara, Dalian, China) as the manufacturer’s protocol. An ABI-Prism 7500 sequence detection system (Applied Biosystems, Foster City, CA, USA) performed RT-qPCR procedures. The PCR reaction volume was 10 µL. The PCR conditions were an initial denaturation step at 95 °C for 30 s, followed by 40 cycles at 95 °C for 5 s and annealing and extension temperature at 60 °C for 34 s. The relative mRNA levels of *Claudin-1*, *ZO-1*, *AQP3*, *Mucin2*, *TNF-α*, *IL-10*, *TGF-β*, *NOD1*, and *MYD88* were calculated by the 2^−∆∆Ct^ method and were normalized by the expression of the housekeeping gene GAPDH. Primer sequences used in the present study are shown in Table 2. Primers were obtained from Sangon Biotech Co., Ltd. (Shanghai, China).

### 2.10. Ileal Microbiota Analysis

The *E. coli* O78 was the most commonly isolated from clinical field, which usually colonized in ileum [20]. The *E. coli* O2 showed special affinity for the jejunum, and O1 has a relatively broad colonization range in the intestine of broilers. Therefore, the ileal microbiota were analyzed. The bacterial DNA was extracted from the ileal digesta using a QIAamp DNA Stool Mini Kit (Qiagen Inc., Valencia, CA, USA). The integrity of DNA was verified by agarose gel electrophoresis. The qualified DNA was used as a template for PCR amplification with 341F and 805R primers (5′-CCTACGGGNBGCASCAG-3′ and 5′-GACTACNVGGGTATCAATCC-3′) targeting the variable V3–V4 gene region. Purified PCR amplification products were used to construct sequencing libraries on a HiSeq PE250 (Illumina, CA, USA). Paired-end reads were de-multiplexed and quality-filtered by Trimmomatic (version 0.36) and then merged by Flash (version 1.2.11). The sequences were clustered into operational taxonomic units (OTUs) with 97% similarity using UPARSE (version 7.1). The taxonomy of each OTU representative sequence was analyzed by the SILVA database (v138). The ileal microbial DNA extraction, amplification, and sequencing were performed by Novogene Technology Co., Ltd. (Beijing, China). Analysis of alpha diversity was performed using Mothur software (version 1.30.1). The Venn diagram was drawn with R (Version 3.0.3). Microbial abundance at different taxonomic levels were generated using Qiime software (Qiime2-2019.7). LEfse software (Version 1.0) was used to perform LEFse analysis, with LDA score > 3. The R (Version 2.15.3) was used to implement the *t*-test analysis with *p* value < 0.05. The PICRUSt analysis was used to predict the functional potential of bacteria communities. The OTUs were normalized by copy number, and metagenome prediction was further categorized into Kyoto Encyclopedia of Genes and Genomes (KEGGs).

### 2.11. Statistical Analysis

Data from this trial were analyzed by one-way ANOVA using SPSS Statistics 26.0. Duncan’s multiple range test was used for intergroup comparisons. The mortality and intestinal histologic scores were analyzed by the Chi-square test. Data are presented as mean ± standard deviation (SD). The “*p* < 0.05” indicated a significant difference.

## 3. Results

### 3.1. Growth Performance

As shown in Table 3, dietary LR supplementation significantly increased the average daily gain (ADG) compared with the *E. coli*-challenged group during days 1–18 (*p* < 0.05). However, no differences were found in ADFI, FCR, and mortality among treatments (*p* > 0.05).

### 3.2. Intestinal Histological Scoring, Permeability and Morphology

**Figure 1:**
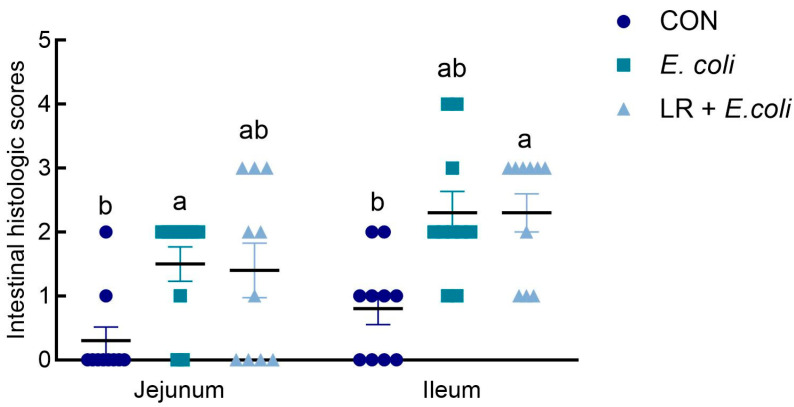
Effects of dietary *Lactobacillus reuteri* postbiotic (LR) supplementation on histologic scoring in jejunum and ileum. Data are presented as mean ± SE (*n* = 10). ^a,b^ Groups with different superscripts differ significantly (*p* < 0.05). CTR, control group; *E. coli*, *E. coli*-challenged group; LR + *E. coli*, *E. coli*-challenged group supplemented with LR.

As exhibited in Figure 1, compared with the CON group, *E. coli* challenge significantly increased histologic score in the jejunum (*p* < 0.05), and histologic score in LR + *E. coli* group did not differ from the other two groups (*p* > 0.05). In the ileum, the LR + *E. coli* group had higher histologic score than that of the CON group (*p* < 0.05).

As shown in Table 4, the intestinal permeability was detected by determining the concentration of DAO in the serum of broilers. There were no differences in DAO levels among treatments (*p* > 0.05). The *E. coli* infection and LR supplementation also did not significantly affect jejunal and ileal morphology (*p* > 0.05).

### 3.3. Expression of Intestinal Barrier-Related Genes in Jejunal and Ileal Mucosa

**Figure 2:**
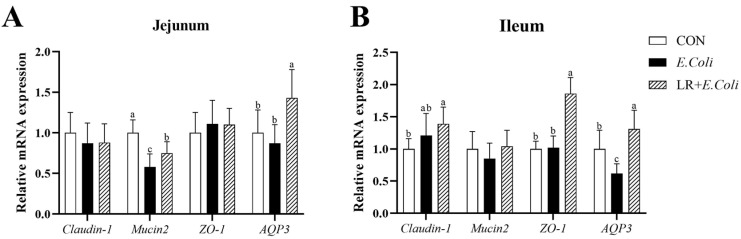
Effects of dietary *Lactobacillus reuteri* postbiotic (LR) supplementation on the relative mRNA expression of intestinal barrier-related genes in the (**A**) jejunal and (**B**) ileal mucosa of broilers challenged with *Escherichia coli*. Data are presented as mean ± SD (*n* = 12). ^a–c^ Bars with different superscripts differ significantly (*p* < 0.05). CTR—control group; *E. coli*—*E. coli*-challenged group; LR + *E. coli*—*E. coli*-challenged group supplemented with LR. *ZO-1*—zonula occludens 1; *AQP3*—aquaporin-3.

Figure 2 shows the relative mRNA expression levels of genes related to intestinal barrier (*Claudin-1*, *Mucin2*, *ZO-1* and *AQP3*) in the jejunal and ileal mucosa of broilers. The *E. coli* challenge down-regulated the mRNA expression of *Mucin2* (*p* < 0.05) in jejunal mucosa and *AQP3* (*p* < 0.05) in ileal mucosa compared with the control group. Birds fed the LR diet exhibited higher mRNA expression of *AQP3* (*p* < 0.05) in the jejunal and ileal mucosa than those in the other groups. A similar effect on the mRNA abundance of *ZO-1* and *Claudin-1* in the ileal mucosa was observed. Furthermore, dietary LR addition improved the relative mRNA expressions of *Mucin2* (*p* < 0.05) in the jejunal mucosa of birds under the *E. coli* challenge.

### 3.4. Intestinal Antioxidant Capability

**Figure 3:**
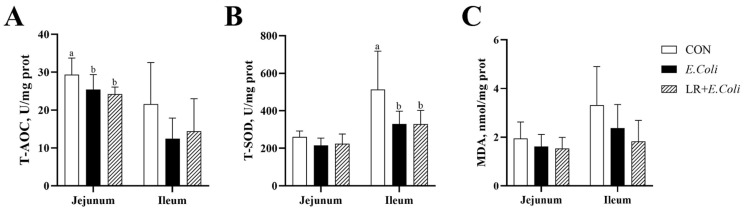
Effects of dietary *Lactobacillus reuteri* postbiotic (LR) supplementation on intestinal antioxidant capacity in *Escherichia coli*-challenged broilers. The concentrations of malondialdehyde (MDA) (**A**) and the activities of total superoxide dismutase (T-SOD) (**B**) and total antioxidant capacity (T-AOC) (**C**) in the jejunal and ileal mucosa are shown. Data are presented as mean ± SD (*n* = 6). ^a,b^ Bars with different superscripts differ significantly (*p* < 0.05). CTR—control group; *E. coli*—*E. coli*-challenged group; LR + *E. coli*—*E. coli*-challenged group supplemented with LR.

The concentration of MDA, T-AOC, and T-SOD in the jejunal and ileal mucosa was assessed to determine the oxidative status in broilers (Figure 3). The infection of *E. coli* decreased the activity of T-AOC (*p* < 0.05) in the jejunal mucosa of broilers. In the ileal mucosa, *E. coli* infection reduced T-SOD activity (*p* < 0.05) in the ileum. However, the dietary LR supplementation failed to increase the intestinal antioxidant capability under the *E. coli* challenge (*p* > 0.05).

### 3.5. Gene Expression of Intestinal Inflammation-Related Cytokine and Pathway

**Figure 4:**
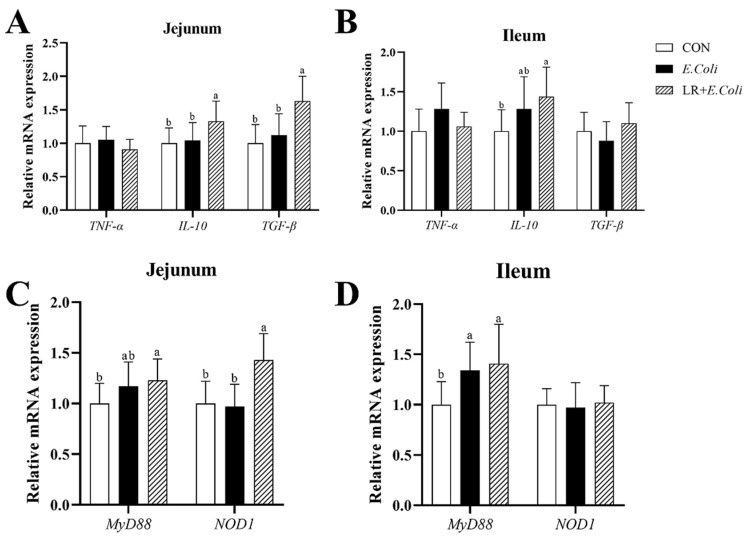
Effect of dietary LR supplementation on relative mRNA expression of inflammation-related pathway and cytokine genes in jejunal and ileal mucosa of *E. coli*-infected broilers. The relative mRNA expression of *TNF-α*, *IL-10* and *TGF-β* in jejunum (**A**) and ileum (**B**) were determined. The relative mRNA expression of *MyD88* and *NOD1* in jejunum (**C**) and ileum (**D**) were determined. ^a,b^ Different superscripts indicate that the difference between the groups was statistically significant (*p* < 0.05), and the data are expressed as mean ± SD. CTR—control group; *E. coli*—the broilers were challenged with *E. coli;* LR + *E. coli*—the challenged broilers were fed a basal diet supplemented with *L. reuteri* postbiotics. Each value represents the mean of 6 replicates per treatment (*n* = 12). Abbreviation: LR—*Lactobacillus reuteri* postbiotics; *TNF-α*—tumor necrosis factor*-α*; *IL-10*—interleukin-10; *TGF-β*—transforming growth factor-β; *MyD88*—myeloid differentiation primary response protein; *NOD1*—nucleotide-binding oligomerization domain containing 1.

The relative mRNA expressions of various inflammatory mediator genes, *TNF-α*, *IL-10*, and *TGF-β*, in the jejunal and ileal mucosa are exhibited in Figure 4. The *E. coli* challenge caused the up-regulation of the mRNA expressions of *TNF-α* and *IL-10* (*p* > 0.05) in the ileal mucosa, with no significant difference found. Birds in the LR group exhibited higher mRNA expressions of *IL-10* (*p* < 0.05) in the jejunal and ileal mucosa, and *TGF-β* in the jejunal mucosa. But no significant difference was found in the mRNA expressions of *TNF-α* in the jejunal and ileal mucosa of birds (*p* > 0.05).

The relative mRNA expressions of inflammation-related signaling pathway NF-κB genes *MyD88* and *NOD1* in the jejunal mucosa are shown in Figure 4. The *E. coli* challenge increased the mRNA expressions of *MyD88* (*p* < 0.05) in the jejunal and ileal mucosa, whereas the addition of LR in diets did not alter the mRNA levels of *MyD88* (*p* > 0.05) compared with *E. coli*-treated chickens. Meanwhile, birds in the LR group showed up-regulated mRNA expression of *NOD1* (*p* < 0.05) in the jejunal mucosa compared with those in the other groups. However, no significant difference was found in the relative mRNA expression of *NOD1* (*p* > 0.05) in the ileal mucosa of birds.

### 3.6. Ileal Microbiota

**Figure 5:**
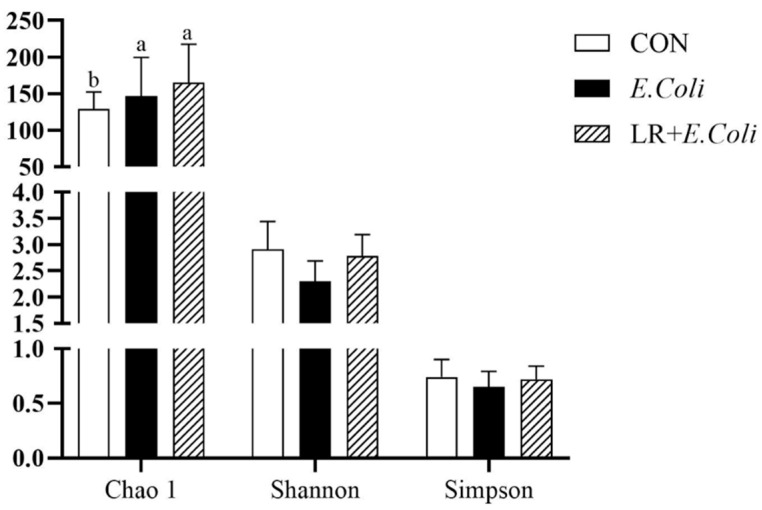
Alpha diversity of the ileal microbiota. The Chao1, Shannon, and Simpson indices are presented. Data are shown as mean ± SD (*n* = 6). ^a,b^ Bars with different superscripts differ significantly (*p* < 0.05). CTR—control group; *E. coli*—*E. coli*-challenged group; LR + *E. coli*—*E. coli*-challenged group supplemented with *Lactobacillus reuteri* postbiotics (LR).

The alpha diversity of the ileal microbiota is shown in Figure 5. The *E. coli* challenge significantly increased the Chao 1 index (*p* < 0.05). However, no significant differences were observed in the Shannon and Simpson indices among the groups (*p* > 0.05).

**Figure 6:**
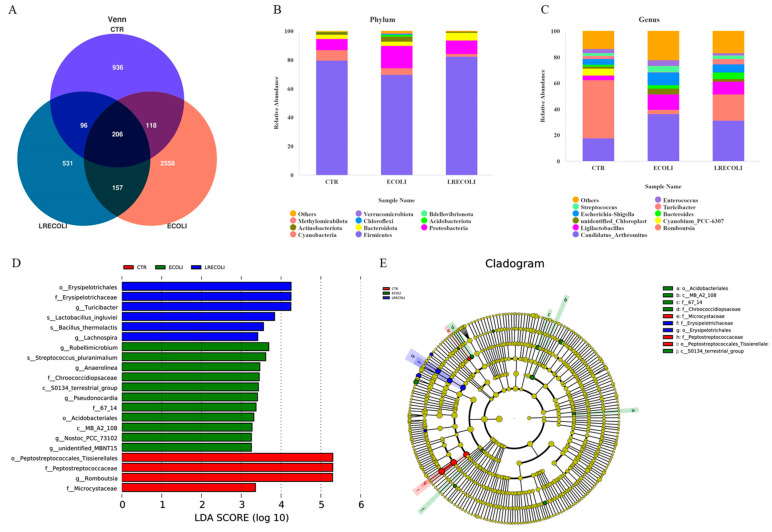
Several significantly differential bacteria in ileum. (**A**) Venn diagram of community analysis, (**B**) phylum level and (**C**) genus level microbiome bar graph, and (**D**) LEfSe and (**E**) LEfSe tree analysis. CTR—control group; ECOLI—the broilers were challenged with *E. coli;* LRECOLI—the challenged broilers were fed a basal diet supplemented with *L. reuteri* postbiotics. Each value represents the means of 6 replicates per treatment (*n* = 6).

Based on 97% sequence similarity, Tags were clustered into 4602 OTUs, of which three groups shared 206 OTUs, and only 936, 2558, and 531 OTUs were exclusive in CTR, *E. coli*, and *E. coli +* LR groups, respectively (Figure 6A). The most abundant (top 10) phyla and genus of cecal microbiota are presented in Figure 6. At the phylum level (Figure 6B), the ileal microbiota was dominated by Firmicutes, Proteobacteria, Cyanobacteria, and Bacteroidota. The *E. coli* challenge increased the relative abundance of Proteobacteria and Actinobacteriota and decreased the relative abundance of Firmicutes and Cyanobacteria. Moreover, dietary LR alleviated the decrease in the relative abundance of Firmicutes and the increase in relative abundance of Proteobacteria and Actinobacteriota. At the genus level (Figure 6C), the *E. coli* challenge increased the relative abundance of *Candidatus_Arthromitus*, *Escherichia-Shigella*, *Ligilactobacillus*, and *Streptococcus* and decreased the relative abundance of *Romboutsia* and *Cyanobium_PCC-6307*. Compared with the *E. coli*-challenged group, dietary supplementation with LR reshaped the ileal flora via elevating the relative abundance of *Romboutsia* and *Bacteroidota* and dropped it of *Candidatus_Arthromitus* and *Escherichia-Shigella*.

**Figure 7:**
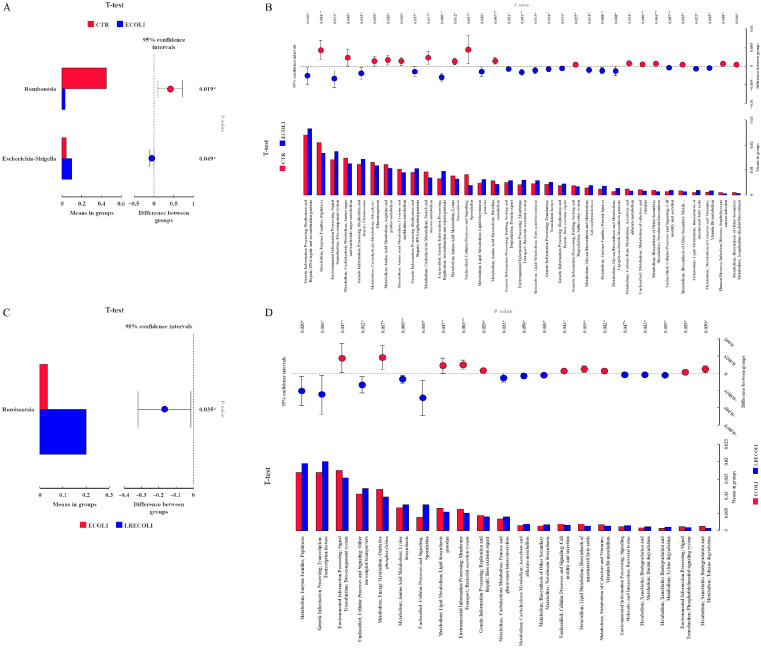
The *t*-test and mean proportion in predicted pathways of the ileal microbiota in various treatments. (**A**) *t*-test is a horizontal analysis, (**B**) *t*-test species level analysis, (**C**) functional prediction of CTR vs. *E. coli*, and (**D**) *E. coli* vs. LR + *E. coli*. * *p* < 0.05; ** *p* < 0.01. CTR—control group; ECOLI—the broilers were challenged with *E. coli;* LRECOLI—the challenged broilers were fed a basal diet supplemented with *L. reuteri* postbiotics. Each value represents the mean of 6 replicates per treatment (*n* = 6).

LEfSe analysis (Figure 6D,E) was used to identify biomarkers with statistically significant differences between groups, and the outcomes demonstrated that *g_Romboutsia* was regarded as the dominant bacteria in the control group. In contrast, *g_Rubellimicrobium* and *s_Streptococcus pluranimalium* were regarded as the dominant bacteria in the *E. coli* group. Dietary supplementary with LR helped to enrich more *s_Lactobacillus_ingluviei*, *s_Bacillus_thermolactis*, and *g_Lachnospira*. As analyzed at the dependent level of *t*-test (Figure 7A), the ileal microbiota in the *E. coli* group possessed more enriched *Escherichia_Shigella* and less *Romboutsia* (*p* < 0.05). Compared with the *E. coli* group (Figure 7C), dietary addition of LR had a higher abundance in *Romboutsia* (*p* < 0.05).

All alterations in the presumptive function were evaluated using PICRUSt in the ileal microbiota at 19 days of the broilers. The *t*-test results in functional prediction are shown in Figure 7B,D. The *E. coli* challenge significantly influenced the abundance of 33 functional pathways (*p* < 0.05), including genetic information processing, metabolism, environmental information processing, etc. Meanwhile, there were significant differences in the abundance of 21 functional pathways (*p* < 0.05) between the *E. coli*-challenged group and the LR treatment. Dietary LR treatment showed a significantly larger abundance of metabolism, genetic information processing, and cellular processes and signaling compared with *E. coli* group.

## 4. Discussion

Avian pathogenic *E. coli* (APEC) is a major cause of substantial economic losses in the poultry industry. This pathogen poses a significant challenge, as it causes a prevalent intestinal disease in broilers that impairs growth traits [21]. Growth performance is the most comprehensive indicator of chicken production efficiency. Previous studies reported that the *E. coli* challenge reduced body weight, ADFI, and ADG, while heightening FCR and broilers’ feed intake [4,22]. In this study, *E. coli* infection decreased growth performance without statistical significance, which is consistent with another study [6]. Moreover, postbiotics could be used as a potential alternative antibiotic growth promoter and anti-stress treatment in the poultry industry [23]. The addition of dietary postbiotics in chicks resulted in higher final body weight, body weight gain, and lower FCR [24]. Supplementation with postbiotics in the diet significantly reduced diarrhea incidence and promoted growth performance in weaned piglets [13]. In the present study, dietary supplementation of LR significantly increased ADG of *E. coli*-infected broilers during days 1–18. Similar results were observed in previous report [25].

Barrier function plays a crucial role in broiler health and performance, mainly referring to the intestinal barrier, which consists of a physical barrier, a chemical barrier, and an immune barrier that work together to prevent pathogens from entering the circulatory system, thus protecting broilers from infection and disease [26]. A healthy intestinal barrier promotes nutrient absorption and increases feed conversion, thereby improving broiler growth performance and productivity. The DAO is an intracellular enzyme in the small intestinal epithelia, which will be released into the peripheral circulation under gut injury, so their blood concentrations could reflect the integrity of the intestinal mechanical barrier and the degree of intestinal mucosal damage [27]. In the present study, serum DAO was not significantly affected by *E. coli* challenge and dietary LR. This indicated that mild impairment of the intestinal barrier did not induce intestinal permeability.

Tight junctions are essential for maintaining the epithelial physical barrier, preventing macromolecule transmission [28]. ZO-1, mucin2, and claudin-1 are the most critical proteins for the intestinal barrier. As many studies reported [29,30], the infection of *E. coli* can reduce the mRNA expressions of *ZO-1*, *Mucin2*, and *Claudin-1* in broilers through the activation of the NF-κB signaling pathway. In this study, LR addition in the broiler diet exhibited greater attenuation of gut impairment, along with increased *Mucin2* and *ZO-1* expression. Similarly, dietary supplementation of *Lactobacillus reuteri* can improve intestinal barrier function via increasing tight junction protein transcripts in the mucosa of the jejunum and ileum, and inhibiting the reduction in tight junction protein expression induced by lipopolysaccharide [31]. Additionally, LR treatment in the broiler diet alleviated the reduced mRNA expression of *AQP3* in the jejunal and ileal mucosa under *E. coli* infection. The aquaporin (AQP) proteins are key in controlling cell volume and water homeostasis [32]. The *E. coli* challenge can down-regulate the transcription level of ileal *AQP3* expression in broilers [33], which is in line with our result. The supplement of dietary LR alleviated the decrease in *AQP3* mRNA expression induced by *E. coli* challenge.

A series of studies have reported that the *E. coli* challenge can activate NF-κB signaling pathways and subsequent inflammatory response and oxidative stress in broilers [29,30]. Moreover, NF-κB signaling pathways are associated with the impaired intestinal barrier dysfunctions [30,34]. In the present study, the *E. coli* challenge elevated mRNA expression of *MyD88* in the ileal mucosa, while no significant changes in proinflammatory gene (*IL-10*, *TGF-β*, and *TNF-α*) expression were found. Interestingly, the mRNA expressions of *MyD88*, *NOD1*, *IL-10*, and *TGF-β* in the jejunal mucosa were increased in the broilers only fed the diet with LR supplementation. Therefore, administration of LR might enhance chickens’ immunocompetency by increasing cytokine production [35]. In addition, the *E. coli* challenge decreased T-AOC activity in the jejunal mucosa and T-SOD in the ileal mucosa. This means that the E. coli challenge may trigger intestinal epithelium injury and a disorder of mucosal barrier function by enhancing the oxidative stress of broilers.

The high diversity of intestinal microbiota to the host is beneficial to enhancing growth performance, maintaining mucosal barrier function, and defending against the invasion of pathogenic microorganisms [28,36]. Dominant bacteria and their metabolic products play a positive role here. A study demonstrated that dietary supplementary with postbiotics had positive effects on microbiota by supporting the increase in beneficial microbes like the *Firmicutes* while decreasing harmful microbes like the *Proteobacteria* [37]. Another study also contributed to keeping the homeostasis of the intestinal flora structure in birds challenged with *Clostridium perfringens* by reshaping the relative abundance of *Firmicutes*, *Proteobacteria*, and *Bacteroidetes* [38]. In the present study, both *E. coli* infection and LR treatment increased the Chao 1 index of the intestinal flora, while they had no effect on the other α-diversity index. This indicated that the treatment with exogenous bacteria or probiotic preparations could increase the richness of a stable flora, while this was not sufficient to change its diversity. We were willing to believe that the proportion of the phylum *Proteobacteria* and the genus *Escherichia-Shigella* was closely related to the inflammation in broilers [39]. The relative amounts of *Escherichia-Shigella* are correlated negatively with weight gain, fecal fat digestibility, intestinal antioxidation, and morphology of broilers [40,41]. This may explain why *Escherichia-Shigella* are associated with adverse effects on the production performance of animals. In the present study, the relative abundance of *Escherichia-Shigella* was enriched in the *E. coli*-treated group, and the intestinal function of broilers in this group also suffered the injury as mentioned above. These results should seem logically consistent. Interestingly, the beneficial effect of LR on intestinal health might be that it down-regulated the relative abundance of *Escherichia-Shigella* in the intestine of birds challenged with *E. coli*.

To gain further insight into the influence of LR on the intestinal microbiota structure, LEfSe and *t*-test analyses showed that dietary supplementation with LR reshaped the ileal flora via elevating the relative abundance of *Romboutsia* and *Lactobacillus_ingluviei* as well as dropped it of *Escherichia-Shigella*. The beneficial effects of *Lactobacillus* on intestinal health seemed to have been widely recognized. The importance of *Romboutsia* for broiler intestinal function has been demonstrated in many studies; for instance, the average abundance of the genera *Bacteroides* and *Romboutsia* in the cecal digesta was positively correlated with body weight and average daily gain [42]. Previous studies also found that *Romboutsia lituseburensis* was closely related to the immune system development of broiler chickens [43,44]. Although in-depth mechanistic studies were beyond the scope of this study, our results suggest that the improvement of intestinal health in broilers by LR might be related to its remodeling of *Romboutsia* and *Escherichia-Shigella*. Changes in the intestinal flora will inevitably lead to differences in metabolic levels and function. In the present study, dietary supplementation with LR reshaped 21 functional pathways of the 33 functional pathways influenced by *E. coli* treatment, which might explain the significant intestinal inflammation between dietary LR and *E. coli* challenge in our results.

## 5. Conclusions

Dietary supplementation with LR up-regulated the expression of genes involving intestinal barrier and anti-inflammation, such as *Mucin2*, *ZO-1*, *AQP3*, *IL-10*, and *TGF-β*, and reshaped the intestinal flora in *E. coli*-challenged broilers. These findings demonstrated the potential of *Lactobacillus* postbiotics in supporting intestinal microbiota under pathogenic challenge.

## Figures and Tables

**Table 1:** Basal diet composition and nutrient levels (dry matter basis).

Ingredient, %	Content (%)	Nutrient and Energy Composition ^3^	Content (%)
Maize	51.73	Metabolizable energy/(MJ/kg)	12.22
Soybean meal	40.73	Crude protein	21.50
Soybean oil	3.36	Lysine	1.17
Calcium hydrogen phosphate	1.92	Threonine	0.82
Limestone	1.16	Calcium	1.00
Salt	0.35	Available phosphorus	0.45
DL-Methionine	0.26	Methionine + Cystine	0.90
Choline chloride (50%)	0.25		
Mineral premix ^1^	0.20		
Vitamin premix ^2^	0.04		
Total	100.00		

^1^ Trace element premix (provided per kilogram of feed) included the following substances: Mn, 100 mg; Zn, 75 mg; Fe, 80 mg; Cu, 8 mg; I, 0.35 mg; Se, 0.15 mg. ^2^ Vitamin premix (provided per kilogram of feed) included the following substances: vitamin A, 12500 IU; vitamin D_3_, 2500 IU; vitamin K_3_, 2.65 mg; vitamin B_1_, 2 mg; vitamin B_2_, 2 mg; vitamin B_12_, 0.025 mg; vitamin E, 30 IU; biotin, 0.0325 mg; folic acid, 1.25 mg; pantothenic acid, 12 mg; nicotinic acid, 50 mg. ^3^ Calculated values.

**Table 2:** List of gene primer sequences.

Name	Accession Number	Sequencer (5′→3′)	Product Length (bp)
*Claudin-1*	NM_001013611.2	F: AAGTGCATGGAGGATGACCA R: GCCACTCTGTTGCCATACCA	119
*ZO-1*	XM_040680632.1	F: TATGAAGATCGTGCGCCTCC R: GAGGTCTGCCATCGTAGCTC	131
*Mucin2*	XM_421035	F: TTCATGATGCCTGCTCTTGTG R: CCTGAGCCTTGGTACATTCTTGT	93
*AQP3*	XM_046936218.1	F: CTTTGCTGTGATGCTTGGCA R: CTGGTAGCTTGATCCAGGGC	116
*TNF-α*	NM_204267.2	F: GAGCGTTGACTTGGCTGTC R: AAGCAACAACCAGCTATGCAC	64
*IL-10*	NM_001004414.4	F: CGCTGTCACCGCTTCTTCA R: CGTCTCCTTGATCTGCTTGATG	63
*TGF-β*	NM_001031045.4	F: TCATCACCAGGACAGCGTTA R: TGTGATGGAGCCATTCATGT	109
*NOD1*	XM_046923314.1	F: AGCACTGTCCATCCTCTGTCC R: TGAGGGTTGGTAAAGGTCTGCT	214
*MYD88*	NM_001030962	F: CTGGCATCTTCTGAGTAGT R: TTCCTTATAGTTCTGGCTTCT	76
*GAPDH*	NM_204305.2	F: CCTAGGATACACAGAGGACCAGGTT R: GGTGGAGGAATGGCTGTCA	64

*ZO-1*—zonula occludens 1; *TNF-α*—tumor necrosis factor-α; *NOD1*—nucleotide-binding oligomerization domain containing 1; *IL-10*—interleukin-10; *TGF-β*—transforming growth factor-β; *MyD88*—myeloid differentiation primary response protein; *AQP3*—aquaporin-3; *GAPDH*—glyceraldehyde-3-phosphate dehydrogenase.

**Table 3:** Effects of LR on growth performance of *E. coli*-infected broilers.

Items	CON	*E. coli*	*LR* + *E. coli*	*p* Values
BW (g)				
d 18	508.41 ± 5.68	499.25 ± 9.21	505.61 ± 10.48	0.242
d 28	1176.33 ± 27.89	1132.49 ± 53.69	1179.25 ± 13.59	0.069
d 1–18				
ADFI (g)	36.43 ± 0.8	35.97 ± 0.21	37.13 ± 1.11	0.069
ADG (g)	25.84 ± 0.31 ^ab^	25.34 ± 0.46 ^b^	26.19 ± 0.65 ^a^	0.029
FCR	1.41 ± 0.02	1.42 ± 0.03	1.42 ± 0.03	0.773
Mortality	3.33% (2/58)	5.00% (3/57)	1.67% (1/59)	0.596
d 19–28				
ADFI (g)	114.48 ± 5.22	111.33 ± 3.49	112.91 ± 1.71	0.372
ADG (g)	67.87 ± 3.67	62.8 ± 4.81	67.01 ± 1.42	0.059
FCR	1.69 ± 0.12	1.78 ± 0.12	1.69 ± 0.05	0.225
Mortality	2.08% (1/47)	6.25% (3/45)	2.08% (1/47)	0.437
d 1–28				
ADFI (g)	69.80 ± 3.04	69.55 ± 2.28	72.85 ± 4.35	0.196
ADG (g)	40.47 ± 1.00	38.90 ± 1.92	40.58 ± 0.48	0.069
FCR	1.73 ± 0.07	1.79 ± 0.09	1.80 ± 0.12	0.370
Mortality	5.00% (3/57)	10.00% (6/54)	3.33% (2/58)	0.284

The data were expressed as mean ± SD. CTR—control group; *E. coli*—the broilers were challenged with *E. coli*; LR + *E. coli*—the challenged broilers were fed a basal diet supplemented with *L. reuteri* postbiotics. Each value represents the means of 6 replicates per treatment (*n* = 6). ^a,b^ Means with different superscripts differ significantly (*p* < 0.05). Abbreviation: LR—*Lactobacillus reuteri* postbiotics; BW—body weight; ADFI—average daily feed intake; ADG—average daily gain; FCR—feed conversion ratio, ADFI/ADG. Mortality was presented as the ratio of dead bird number to all birds in each group (dead bird number/alive bird number).

**Table 4:** Effect of dietary LR on serum activity of diamine oxidase and intestinal morphology in *E. coli*-infected broilers at day 19.

Item	CTR	*E. coli*	LR + *E. coli*	*p* Values
Serum DAO (U/L)	15.84 ± 5.56	20.56 ± 9.73	16.87 ± 4.76	0.385
Jejunum				
VH (μm)	1039.15 ± 141.91	1075.72 ± 170.05	1039.16 ± 200.97	0.862
CD (μm)	145.61 ± 29.57	158.10 ± 33.49	139.50 ± 29.90	0.406
VH/CD	7.48 ± 1.05	7.12 ± 0.89	7.87 ± 1.47	0.366
Ileum				
VH (μm)	711.75 ± 152.76	775.14 ± 134.24	666.78 ± 60.19	0.287
CD (μm)	135.41 ± 30.48	154.64 ± 44.76	140.57 ± 35.16	0.529
VH/CD	5.45 ± 0.53	5.27 ± 0.71	5.07 ± 0.81	0.556

The data are expressed as mean ± SD. CTR, control group; *E. coli*, the broilers were challenged with *E. coli;* LR + *E. coli*, the challenged broilers were fed a basal diet supplemented with *L. reuteri* postbiotics. Each value represents the mean of 6 replicates per treatment (*n* = 10). Abbreviation: LR—*Lactobacillus reuteri* postbiotics; DAO—diamine oxidase.

## Data Availability

The datasets produced and/or analyzed during the current study are available from the corresponding author upon reasonable request.
